# Supplementary material for: Troxerutin flavonoid has neuroprotective properties and increases neurite outgrowth and migration of neural stem cells from the subventricular zone
Source: PLoS One. 2020 Aug 14;15(8):e0237025. doi: 10.1371/journal.pone.0237025 (PMC7428079; doi:10.1371/journal.pone.0237025)
Supplement: S1 File — (DOCX) [file pone.0237025.s001.docx]

**Supplementary material**

**Calcein/Propidium iodide Live-dead assay**

Calcein/propidium live-dead assay was performed to evaluate if test concentrations of troxerutin flavonoid show cytotoxicity in NSCs culture. Around 10000 NSCs were proliferated in DMEM /F-12 differentiation medium into each well of a 96 well plate precoated With ECM gel for 48 h with and without exposure to 25 µM, 50 µM and 100 µM concentrations of troxerutin. The live-dead assay was performed in the same way as described by the researchers in their protocol ([1](#_ENREF_1)). The live-dead reagent contains Calcein-AM (C3100MP, Life Technology, Eugene, USA) as a marker for viable cells and propidium iodide (P1304MP, Thermofischer, Waltham, USA). Calcein is a colourless compound and easily permitted through the cell membrane of viable cells. With in the living cell, calcein is converted into a fluorescent marker and gives green fluorescence. Propidium iodide enters only enter through the broken cell membrane of dead cells and is intercalated into the nucleic acid and gives red fluorescence. At least five images of independent microscopic fields were capture for each of phase contrast, green and red fluorescent modes by a 20 x objective of a fluorescent microscope (Olympus Corporation, Tokyo, Japan) for each condition in each replicate. Percentage of calcein +ve viable cells was obtained from the total cell count in the phase-contrast images. The experiment was performed as five replicates.

**WST-1 cytotoxicity assay**

Cytotoxicity of 10µM Aß42 was assessed through WST-1 cytotoxicity assay. Around 40000 NSCs were differentiated into each well of a 96-well plate pre-coated with PDL for 48 h in the presence of Aß42. Control was the vehicle used for making Aß42 working solution. WST-1 assay was performed by following the Kit method (WST-1, Roche, Mannheim, Germany). Photometric absorbance was measured using a GENios fluorescent multi-plate reader (TECAN, Austria).

**Supporting results and figure.**

We found a non-significant difference between the control and all the tested concentrations of troxerutin concerning the viable percentage of NSCs after 7 days exposure (Fig SA). WST-1 assay revealed that 10µM Aß42 insignificantly affected the viability of neurons/astrocytes co-culture differentiated for 48 h (Fig SB).


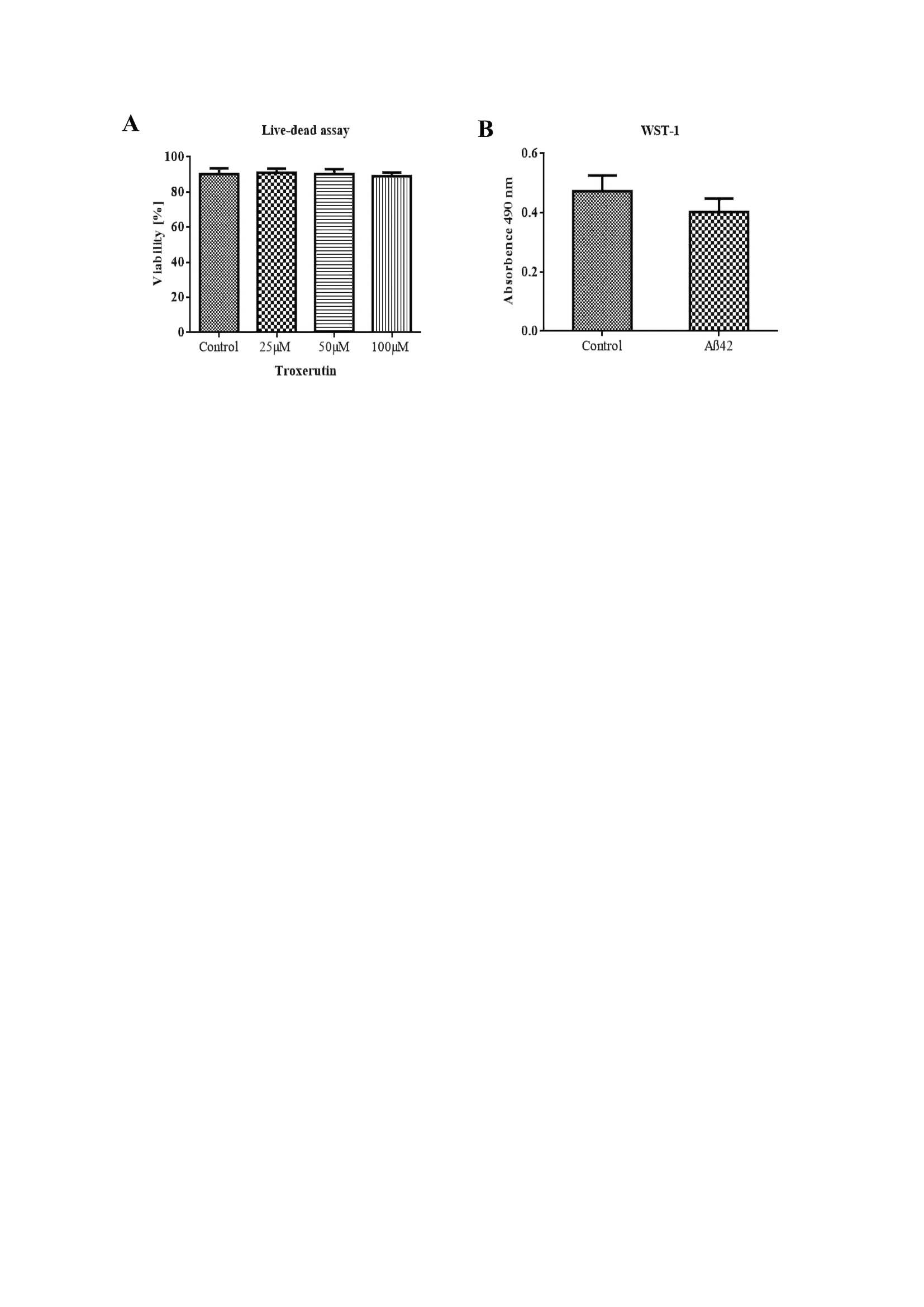


**Fig S. Live-dead and WST-1 cytotoxicity assays.** Calcein/propidium iodide live-dead assay was performed for three test concentrations of TRX. NSCs were exposed to TRX for 48 h followed by staining with Calcein AM as a live cell marker and propidium iodide as a dead cell marker. **A:** Represents the percentage of viable cells which was calculated from the total cell count. WST-1 was performed for differentiated culture of NSCs for 48 h exposed to 10µM Aß42. Test was performed in a 96 well plate. **B:** Represents the spectrophotometer absorbance values. Con; Control, TRX; troxerutin. Data are presented as mean±SEM. Experiments were performed as 5 replicates (n=5).

Moreover, a non-significant difference concerning NSCs viability was also observed betweenthe Control and 100µM TRX.

**Supplementary raw data**

Values in each table are from 5 independent experiments and are the values behind the bar graph.

**Fig 2.** TRX effect on neural stem cell markers and proliferation of NSCs.

| **Fig.2(A)** | Neurosphere Number | | |  |  |  |  |  |  |  |  |
| --- | --- | --- | --- | --- | --- | --- | --- | --- | --- | --- | --- |
| 3rd day | | | | 5th day | | | | 7th day | | | |
| Con | 25µM | 50µM | 100µM | Con | 25µM | 50µM | 100µM | Con | 25µM | 50µM | 100µM |
| 59 | 60 | 69 | 62 | 160 | 135 | 149 | 142 | 114 | 116 | 115 | 104 |
| 88 | 112 | 98 | 96 | 148 | 157 | 162 | 146 | 142 | 146 | 141 | 136 |
| 62 | 64 | 71 | 64 | 136 | 142 | 134 | 121 | 106 | 111 | 117 | 116 |
| 77 | 70 | 86 | 83 | 119 | 121 | 140 | 137 | 113 | 108 | 109 | 97 |
| 75 | 83 | 78 | 85 | 139 | 130 | 135 | 129 | 108 | 102 | 119 | 123 |

| **Fig.2(B)** | Neurosphere Diameter | | |  |  |  |  |  |  |  |  |
| --- | --- | --- | --- | --- | --- | --- | --- | --- | --- | --- | --- |
| 3rd day | | | | 5th day | | | | 7th day | | | |
| Con | 25µM | 50µM | 100µM | Con | 25µM | 50µM | 100µM | Con | 25µM | 50µM | 100µM |
| 34 | 35 | 36 | 35 | 55 | 56 | 54 | 56 | 90 | 87 | 85 | 104 |
| 38 | 37 | 36 | 37 | 57 | 57 | 58 | 61 | 87 | 88 | 84 | 89 |
| 35 | 35 | 34 | 35 | 65 | 62 | 64 | 63 | 93 | 89 | 84 | 88 |
| 36 | 35 | 35 | 36 | 66 | 66 | 62 | 64 | 89 | 87 | 90 | 88 |
| 35 | 36 | 35 | 35 | 64 | 64 | 64 | 66 | 93 | 88 | 85 | 85 |

| **Fig.2(C)** |  | | |  |  |  |  |  |  |  |  |
| --- | --- | --- | --- | --- | --- | --- | --- | --- | --- | --- | --- |
| BrdU | | | | Ki67 | | | | BrdU/Ki67 | | | |
| Con | 25µM | 50µM | 100µM | Con | 25µM | 50µM | 100µM | Con | 25µM | 50µM | 100µM |
| 52 | 49,7 | 50 | 53 | 66 | 68 | 72 | 70 | 67 | 66 | 65 | 67 |
| 54 | 52 | 52,8 | 54 | 69 | 74,8 | 76 | 77 | 69 | 69 | 68 | 67,7 |
| 55,2 | 54,8 | 57,6 | 54 | 74 | 75 | 76 | 77 | 70,7 | 69 | 69 | 68 |
| 55,6 | 56 | 59 | 56 | 77 | 78,9 | 77 | 80 | 74,8 | 69 | 70 | 69 |
| 56,7 | 56 | 60 | 57,8 | 78 | 80 | 85 | 84 | 81 | 71 | 75 | 70 |

| **Fig.2(D)** |  | |  |  |  |  |  |
| --- | --- | --- | --- | --- | --- | --- | --- |
| Nestin | | | | Nestin.GFAP | | | |
| Con | 25µM | 50µM | 100µM | Con | 25µM | 50µM | 100µM |
| 68,8 | 72 | 72 | 64 | 31 | 27 | 27 | 36 |
| 71 | 74 | 70 | 62 | 28,9 | 25 | 31 | 38 |
| 68 | 71 | 69 | 65 | 32 | 28 | 32 | 43 |
| 68 | 68 | 67 | 53 | 32 | 32 | 29 | 47 |
| 65 | 68 | 71 | 56 | 34 | 31 | 29 | 44 |

| **Fig 4.** TRX effect on NSCs differentiation after 7 days and 48 h of incubation**.** | | | | | | | | | | | | | |
| --- | --- | --- | --- | --- | --- | --- | --- | --- | --- | --- | --- | --- | --- |
| **Fig 4(A)** | |  | |  | |  | |  |  |  | |  | |
| Neurons | | | | | | | | Astrocytes | | | | | |
| Con | | 25µM | | 50µM | | 100µM | | Con | 25µM | 50µM | | 100µM | |
| 41,6 | | 47 | | 45 | | 52 | | 51 | 46,8 | 47,8 | | 45 | |
| 46,5 | | 51,9 | | 52 | | 54 | | 41 | 37 | 36 | | 36,9 | |
| 60,8 | | 64 | | 62,9 | | 67 | | 28 | 25,9 | 27 | | 25 | |
| 48,9 | | 52,8 | | 53 | | 55 | | 40,7 | 38 | 37 | | 35 | |
| 56,9 | | 61 | | 63 | | 68 | | 24 | 22 | 23 | | 23 | |
| **Fig (B)** |  | |  | |  | |  | |  | |  | |  |
| Neurons | | | | | | | Astrocytes | | | | | | |
| Con | 25µM | | 50µM | | 100µM | | Con | | 25µM | | 50µM | | 100µM |
| 56 | 59 | | 61,7 | | 65 | | 30 | | 27 | | 29 | | 25 |
| 36 | 47 | | 50 | | 51,2 | | 41 | | 33,6 | | 38 | | 40 |
| 43,6 | 48 | | 48 | | 49 | | 36 | | 32 | | 36,6 | | 27 |
| 52,9 | 53 | | 59 | | 62,8 | | 27 | | 30 | | 26 | | 32 |
| 56,9 | 56 | | 56 | | 64 | | 25 | | 26,5 | | 28 | | 26 |
| **Fig.4(C)** |  | |  | |  | |  |  |  |  |  |  |  |
| NNN/TN x 100 | | | | | | |  |  |  |  |  |  |  |
| Con | 25µM | | 50µM | | 100µM | |  |  |  |  |  |  |  |
| 4,1 | 2,7 | | 2,7 | | 2,2 | |  |  |  |  |  |  |  |
| 3,3 | 2,6 | | 2,3 | | 2,7 | |  |  |  |  |  |  |  |
| 3,2 | 2,4 | | 3,1 | | 1 | |  |  |  |  |  |  |  |
| 4,7 | 2,9 | | 3,6 | | 2 | |  |  |  |  |  |  |  |
| 9,2 | 6,7 | | 6,1 | | 2,8 | |  |  |  |  |  |  |  |

| **Fig.4(D)** |  |  |  |  |  |  |  |
| --- | --- | --- | --- | --- | --- | --- | --- |
| NNN | | | | Double +ve | | | |
| Con | 25µM | 50µM | 100µM | Con | 25µM | 50µM | 100µM |
| 10 | 6,1 | 4,5 | 3 | 11,4 | 7,1 | 5,6 | 4,53 |
| 10,8 | 7,1 | 8 | 4 | 9,8 | 12,9 | 11,7 | 4 |
| 6,2 | 3,6 | 3,8 | 3 | 10 | 9,7 | 8,4 | 5,9 |
| 5,5 | 2,9 | 2,9 | 3 | 14 | 9,5 | 9,5 | 9,1 |
| 5,7 | 3,4 | 2,99 | 1,84 | 13 | 9,9 | 8,98 | 7,9 |

| **Fig 7.** Effects of TRX on morphological parameters of neurons and astrocytes differentiated from NSCs after 7 days and 48 h of incubation. | | | | | | | | | | | | |
| --- | --- | --- | --- | --- | --- | --- | --- | --- | --- | --- | --- | --- |
| **Fig 7 (A)** |  |  |  |  |  |  |  |  | **Fig 7 (C)** |  |  |  |
| **TN** | | | | **µN** | | | |  | Astrocyte area | | | |
| **Con** | **25µM** | **50µM** | **100µM** | **Con** | **25µM** | **50µM** | **100µM** |  | **Con** | **25µM** | **50µM** | **100µM** |
| 82 | 106 | 106 | 120 | 28 | 38 | 37 | 40 |  | 1870 | 2434 | 2255 | 2195 |
| 79 | 130 | 133 | 160 | 28 | 41 | 45 | 47 |  | 2700 | 3002 | 2715 | 1946 |
| 101 | 149 | 157 | 158 | 36 | 46 | 48 | 48 |  | 2747 | 3041 | 2835 | 1884 |
| 116 | 155 | 150 | 194 | 38 | 49 | 50 | 56 |  | 2361 | 2754 | 2780 | 1952 |
| 96 | 140 | 176 | 167 | 32 | 44 | 45 | 48 |  | 1996 | 2836 | 2719 | 1912 |
|  |  |  |  |  |  |  |  |  |  |  |  |  |
| **Fig 7 (B)** |  |  |  |  | **Fig 7 (D)** |  |  |  |  |  |  |  |
| **TN** | | **µN** | |  | Astrocyte area | |  |  |  |  |  |  |
| **Con** | **100µM** | **Con** | **100µM** |  | **Con** | **100µM** |  |  |  |  |  |  |
| 53,44 | 63,33 | 22,44 | 26,8 |  | 1143 | 1042 |  |  |  |  |  |  |
| 48,84 | 64,5 | 21,6 | 27,4 |  | 1165 | 905 |  |  |  |  |  |  |
| 47,6 | 66,6 | 21,8 | 31,5 |  | 1467 | 778 |  |  |  |  |  |  |
| 52,9 | 67,6 | 24 | 30,8 |  | 1645 | 1055 |  |  |  |  |  |  |
| 49,5 | 60,5 | 21,4 | 27,7 |  | 1601 | 985 |  |  |  |  |  |  |

| **Fig 9.** Effect of TRX on neurite arborisation after 7 days of differentiation. | | | | | | | |  |  |  |  |  |
| --- | --- | --- | --- | --- | --- | --- | --- | --- | --- | --- | --- | --- |
| **Fig 9 (A)** |  |  |  |  |  |  |  |  | **Fig 9 (B)** |  |  |  |
| AP | | | | EP | | | |  | Arborization | | | |
| Con | 25µM | 50µM | 100µM | Con | 25µM | 50µM | 100µM |  | Con | 25µM | 50µM | 100µM |
| 2,3 | 2,38 | 2,45 | 2,53 | 2,82 | 2,9 | 2,99 | 3 |  | 1,22 | 1,21 | 1,22 | 1,18 |
| 2,45 | 2,91 | 2,59 | 2,98 | 2,96 | 3,32 | 3,19 | 3,47 |  | 1,2 | 1,14 | 1,23 | 1,16 |
| 2,35 | 2,4 | 2,46 | 2,29 | 2,87 | 3,26 | 3,37 | 3,27 |  | 1,22 | 1,36 | 1,37 | 1,43 |
| 2,34 | 2,37 | 2,38 | 2,47 | 3 | 3,18 | 3,45 | 3,52 |  | 1,28 | 1,34 | 1,45 | 1,42 |
| 2,92 | 2,87 | 2,47 | 2,66 | 2,92 | 3,35 | 3,42 | 3,65 |  | 1 | 1,16 | 1,38 | 1,37 |

| **Fig 11.** Neuroprotective effects of TRX flavonoid against Aß42 induced depression of differentiation after 48 h of differentiation on ECM coated glass coverslips. | | | | | | | | | | | | | | | |
| --- | --- | --- | --- | --- | --- | --- | --- | --- | --- | --- | --- | --- | --- | --- | --- |
| **Fig 11(A)** | |  |  |  |  |  | **Fig 11(C)** | |  |  |  |  |  |  |  |
| Neurons | | | Astrocytes | | |  | TN | | | µN | | |  |  |  |
| Con | Aß(10µM) | Aß+TRX | Con | Aß(10µM) | Aß+TRX |  | Con | Aß(10µM) | Aß+TRX | Con | Aß(10µM) | Aß+TRX |  |  |  |
| 47,9 | 40 | 60 | 40 | 33,8 | 37,8 |  | 58,49 | 46,49 | 60,15 | 24,58 | 19,84 | 27,23 |  |  |  |
| 46 | 41,7 | 52 | 38 | 35 | 34 |  | 54,84 | 43,39 | 65,78 | 22,94 | 19,03 | 27,29 |  |  |  |
| 45,9 | 38,8 | 50 | 37 | 24 | 29 |  | 55,25 | 44,21 | 66,45 | 22,27 | 20,29 | 28,05 |  |  |  |
| 52,9 | 47 | 48,5 | 29 | 28 | 25 |  | 55,62 | 41,35 | 63,13 | 23,19 | 18,98 | 26,16 |  |  |  |
| 51 | 48,8 | 51 | 36 | 30 | 34 |  | 56,05 | 43,86 | 63,8775 | 23,245 | 19,535 | 27,1825 |  |  |  |
|  |  |  |  |  |  |  |  |  |  |  |  |  |  |  |  |
| **Fig 11(B)** | |  |  |  |  |  | **Fig 11(D)** | |  |  |  |  |  |  |  |
| NNN | | | Double ]ve | | |  | Astrocyte area | | |  |  |  |  |  |  |
| Con | Aß(10µM) | Aß+TRX | Con | Aß(10µM) | Aß+TRX |  | Con | Aß(10µM) | Aß+TRX |  |  |  |  |  |  |
| 14,6 | 29 | 14,2 | 4,9 | 6,4 | 6,6 |  | 1076 | 922 | 946 |  |  |  |  |  |  |
| 12,3 | 41 | 14,7 | 4,9 | 8,3 | 6,5 |  | 1110 | 978 | 856 |  |  |  |  |  |  |
| 13 | 25 | 14,4 | 4,2 | 6,5 | 5 |  | 1182 | 969 | 977 |  |  |  |  |  |  |
| 10 | 36 | 18,6 | 3 | 5,4 | 4,6 |  | 1375 | 911 | 967 |  |  |  |  |  |  |
| 14,9 | 31,5 | 12 | 2,16 | 6,2 | 5,6 |  | 1185,75 | 945 | 936,5 |  |  |  |  |  |  |

| **Fig 14.** TRX enhances the migration of differentiated cells from NSCs cultured for 24 h. | | | | | | | | |
| --- | --- | --- | --- | --- | --- | --- | --- | --- |
|  |  |  |  |  |  |  |  |  |
| Distance travelled by cells | | | |  |  |  |  |  |
| Con | 25µM | 50µM | 100µM |  |  |  |  |  |
| 132 | 129 | 143 | 151 |  |  |  |  |  |
| 126 | 133 | 148 | 172 |  |  |  |  |  |
| 124 | 124 | 154 | 143 |  |  |  |  |  |
| 145 | 131 | 151 | 161 |  |  |  |  |  |
| 110 | 133 | 154 | 145 |  |  |  |  |  |

| **Fig S.** Live-dead and WST-1 cytotoxicity assays. | | | | |  |  |  |
| --- | --- | --- | --- | --- | --- | --- | --- |
|  | **Fig S(A)** |  |  |  |  | **Fig S(B)** |  |
|  | **Viability (%)** | | | |  | Absorbence | |
|  | Con | 25µM | 50µM | 100µM |  | Con | Aß42 |
|  | 89 | 93 | 95 | 91 |  | 0,3533 | 0,3678 |
|  | 86 | 89 | 89 | 89 |  | 0,6026 | 0,515 |
|  | 93 | 93 | 90 | 91 |  | 0,4907 | 0,3474 |
|  | 94 | 88 | 88 | 88 |  | 0,5695 | 0,499 |
|  | 89 | 92 | 89 | 86 |  | 0,347 | 0,2876 |

**References**

1. Sadeh N, Oni-Biton E, Segal M. Acute Live/Dead Assay for the Analysis of Toxic Effects of Drugs on Cultured Neurons. Bio-protocol. 2016; 6:15. doi: 10.21769/BioProtoc.1889.
