# Supplementary material for: Troxerutin flavonoid has neuroprotective properties and increases neurite outgrowth and migration of neural stem cells from the subventricular zone
Source: PLoS One. 2020 Aug 14;15(8):e0237025. doi: 10.1371/journal.pone.0237025 (PMC7428079; doi:10.1371/journal.pone.0237025)
Supplement: Attachment — Submitted filename: Response to reviewer.docx [file pone.0237025.s002.docx]

Zweibrücken, 05.06.2020.

Editor-in-Chief

PLOS ONE

**Response Letter to the manuscript:** PONE-D-20-07816

Dear Sir/Madam,

Thank you very much for sending us the reviewer’s comments on our manuscript.**“Troxerutin Flavonoid has Neuroprotective properties and increases Neurite outgrowth and Migration of Neural stem cells from the Subventricular zone”** Additionally, we would like to thank the reviewers for their encouraging feedback and the decision for giving us the possibility for a revision according to their constructive discussion of weaknesses in our paper.

This response letter summarizes our revisions and addresses the points raised by the reviewers and editor.

Reviewer’s and Editor’s comments are in Bold, reply from the author is in Normal font while informations already present in the first submitted manuscript are in Time New Roman with italic style. Changes to the revised manuscript are in red.

Kind regards

**Comments from Editor:**

**1. Please ensure that your manuscript meets PLOS ONE's style requirements, including those for file naming. The PLOS ONE style templates can be found at** <https://journals.plos.org/plosone/s/file?id=wjVg/PLOSOne_formatting_sample_main_body.pdf> .<https://journals.plos.org/plosone/s/file?id=ba62/PLOSOne_formatting_sample_title_authors_affiliations.pdf>.

**Author’s response**: The revised manuscript including title page has been formatted as per PLOSONe templates.

**2. Thank you for including your ethics statement: 'In the present study Animal preparations were carried out following the guidelines of the institutional ethics committee (University of applied sciences Kaiserslautern) and according to animal protection law in Rhineland-Palatinate state, Germany.'
a. Please amend your current ethics statement to confirm that your named ethics committee specifically approved this study.**

**Author’s Response:** Since in our study none of the experiments was performed directly on the living animals so no separate approval specifically required for this study. Tissues were obtained from the dead animal and animal killing has to be reported to the local Committee on the Ethics of Animal Experiments. University of Applied Sciences Kaiserslautern. The Ethical statement has been amended as per PLOSONe policy.

Following statement has been added to the revised manuscript.

Animal preparations in this study were carried out in strict accordance with the recommendation in the Guide for the care and use of laboratory animals according to animal protection law in Rhineland-Palatinate State, Germany. Since no experiment was directly performed on the living animals and only tissues were taken from the dead animals, no separate approval was necessary and the animal killing only has to be reported to the local Committee on the Ethics of Animal Experiments, University of Applied Sciences Kaiserslautern. (Materials and Methods, Animal dissection and cell culture, Page-5).

**b. Once you have amended this/these statement(s) in the Methods section of the manuscript, please add the same text to the “Ethics Statement” field of the submission form (via “Edit Submission”).**

**Author’s Response:** Amendments regarding the Ethics statement have been done in the submission system of PLOSONe.

**3. Thank you for stating the following in the Financial Disclosure section:'We are very thankful to The University of Applied Sciences, Kaiserslautern, Germany and The Higher Education Commission Government of Pakistan for providing financial support in the execution of the present project.'**

**We note that one or more of the authors are employed by a commercial company: URSAPHARM Arzneimittel GmbH**

**Author’s response:** The statements have been included. The company is involved as a scientific partner with no financial support for this particular study. The company provided Troxerutin as a compound with high water solubility. We performed experiments with conventional rutin and realized that the effect is very poor, due to the reduced solubility. So we were thankful to the company to support us with their product. Peter Meiser, the company’s representative, was also involved in the writing of the paper.

**a. Please provide an amended Funding Statement declaring this commercial affiliation, as well as a statement regarding the Role of Funders in your study. If the funding organization did not play a role in the study design, data collection and analysis, decision to publish, or preparation of the manuscript and only provided financial support in the form of authors' salaries and/or research materials, please review your statements relating to the author contributions, and ensure you have specifically and accurately indicated the role(s) that these authors had in your study. You can update author roles in the Author Contributions section of the online submission**

**Author’s response:** The statement has been amended as per Editor Suggestions. Funding of this study was only received from an internal funding system of the University of Applied Sciences Kaiserslautern, Germany. The commercial company URSA-PHARM Arzneimittel GmbH did not support the study financially but provided the compound Troxerutin and its relevant information. The author’s roles in the author contribution section of the online system have been updated.

Following statement has been added to the coverletter and in the submission system.

These funding agencies provided support in term of stipend for authors and expenses concerning chemicals but did not have any additional role in the study design, data collection and analysis, decision to publish, or preparation of the manuscript. Additionally, Ursapharm Arzneimittel GmbH provided support in the form of salary for author PM, but did not have any additional role in the study design, data collection and analysis, decision to publish, or preparation of the manuscript. The specific roles of these authors are articulated in the ‘author contributions’ section (Updated funding statement, Coverletter KHS).

**b. Please also provide an updated Competing Interests Statement declaring this commercial affiliation along with any other relevant declarations relating to employment, consultancy, patents, products in development, or marketed products, et**

**Author’s response:** Updated competing interest statement has been added to the coverletter, Competing interest form and also uploaded to the submission system.

The authors have read the journal’s policy and have the following potential competing interests: PM is a paid employee of Ursapharm Arzneimittel GmbH. This does not alter our adherence to PLOS ONE policies on sharing data and materials. There are no patents, products in development or marketed products associated with this research to declare.

**c. Please include both an updated Funding Statement and Competing Interests Statement in your cover letter. We will change the online submission form on your behalf**.

**Author’s response:** Both the updated funding statement and competing interest statements have been added to the cover letter.

**4. Please add the concentration or dilution factor of all antibodies used to your Methods section.**

**Author’s response:** The dilution factor of antibodies used has been added to the Methods section of the revised manuscript.

Please find newly added information regarding dilution factor in the revised manuscript and are highlighted in red (Materials and Methods, immunostaining, page-8).

**5. To comply with PLOS ONE submissions requirements, in your Methods section, please provide additional information on the animal research and ensure you have included details on**

**(i) the source and number of mice,**

**Author’s response:**

Animals were housed under specific pathogen-free conditions on a 12 h light/12 h dark cycle according to German regulations in the animal house facilities of the medical faculty Homburg, next to the Zweibrücken Campus. Animals were transported in warmed boxes and killed immediately after arrival by decapitation. For each set of experiment, tissue was removed from a set of 3 animals per experiment. Total of 21 animals was used in this study.

Following information has been added to the revised manuscript.

Animals were housed under specific pathogen-free conditions on a 12 h light/12 h dark cycle according to German regulations in the animal house facilities of the medical faculty Homburg, next to the Zweibrücken Campus. Around three animals were employed used for each set of experiment. The total number of animals used for this study was 21. (Materials and Methods, Animal dissection and cell culture, Page-5).

**(ii) whether anesthesia was used prior to decapitation and, if so, your methods of anesthesia and/or analgesia,**

**Author’s response:** The postnatal animals in our study were killed by decapitation without using anaesthesia.

Following statement has been added to the revised manuscript.

An authorized and well-trained researcher sacrificed the animals by decapitation without anaesthesia. (Materials and Methods, Animal dissection and cell culture, Page-5).

**(iii) whether decapitation was done by trained personnel.**

**Author’s response:** The decapitation was done only by a trained and authorized person.

Following information has been added to the revised manuscript.

An authorized and well-trained researcher sacrificed the animals by decapitation. (Materials and Methods, Animal dissection and cell culture**,** Page-5).

**6. In your Data Availability statement, you have not specified where the minimal data set underlying the results described in your manuscript can be found. PLOS defines a study's minimal data set as the underlying data used to reach the conclusions drawn in the manuscript and any additional data required to replicate the reported study findings in their entirety. All PLOS journals require that the minimal data set be made fully available. For more information about our data policy, please see** [**http://journals.plos.org/plosone/s/data-availability**](http://journals.plos.org/plosone/s/data-availability).

**Author’ response:** Data will be made fully available after acceptance using a data repository.

**7. PLOS requires an ORCID iD for the corresponding author in Editorial Manager on papers submitted after December 6th, 2016. Please ensure that you have an ORCID ID and that it is validated in Editorial Manager. To do this, go to ‘Update my Information’ (in the upper left-hand corner of the main menu), and click on the Fetch/Validate link next to the ORCID field. This will take you to the ORCID site and allow you to create a new iD or authenticate a pre-existing iD in Editorial Manager. Please see the following video for instructions on linking an ORCID ID to your Editorial Manager account:** [**https://www.youtube.com/watch?v=_xcclfuvtxQ**](https://www.youtube.com/watch?v=_xcclfuvtxQ)

**Author’s response:** ORCID ID for the corresponding author has been created. 0000-0002-8763-5484.

**Summarized comments from Editor:**

**i)- potential extracellular effects of your drug on Ab42 toxicity should be controlled,**

**Author’s response:** Determination of extracellular interaction between the troxerutin and Aß42 can be a useful way to understand the basic mechanism of neuroprotective effects of troxerutin against the Aß42 neurotoxicity but at this stage, our intension was to establish the in vitro model for screening biological impact of these molecules and due to the time and budget constraints we did not perform such a type of studies. However, we will consider these studies as a part of our long term future projects.

**(ii) Figure quality has to improve:**

**Author’s replay:** Qualities of all the figures in the revised manuscript have been improved. Fig. 3 has been completely replaced by a new figure as suggested by the reviewer # 1.

Following figure has been added to the revised manuscript

**Fig 3. Neurosphere clonogenic assay for NSCs proliferation.** The medium of Control+ve was supplemented with EGF and FGF growth factors. Growth factors were completely omitted from the Control-ve and all TRX treatments. Cells proliferated rapidly in positive control and formed neurospheres whose size increased with the increase in the incubation period. Cells in all other treatment conditions failed to proliferate and completely died on 7^th^ day of observation. Pictures were captured with a 10 x objective of a phase-contrast microscope. (Results, page-11).

**(iii) Ab42 use has to be better rationalized.**

**Author’s response:** One of the aspects of the present study was to conduct an effect based experiment to evaluate if troxerutin counteracts the inhibitory effects of Aß42 on the short term differentiation of *in vitro* NSCs culture from the postnatal mouse brain. The reason for using Aß42 in the present study was because of its inhibitory effects on NSCs differentiation and also its implication in the leading neurodegenerative disorder Alzheimer’s (1). One of the neurotoxic aspects of Aß42 is the inhibition of neurite growth through mitochondrial dysfunction as shown in an in vitro assay using PC12 cell culture (2). With normal configuration at low concentration, Aß is necessary for regulating the normal functions of the nervous system and also plays a protective role(3). Finally, the concentration of Aß42 selected for our experiment was one reported by researchers as an inhibitory concentration for NSCs differentiation and neurite growth in the hippocampus culture since lower concentration showed neurogenerative rather than neurotoxic effects (4). Additionally, we performed WST-1 viability assay to ascertain if the concentration of the Aß42 we used in our experiment has no direct killing effects rather inhibitory effects on NSCs differentiated culture. We found a non-significant effect on the viability of our culture (Supplementary, **SA Fig)**.

**Fig S. Live-dead and WST-1 cytotoxicity assays.** Calcein/propidium iodide live-dead assay was performed for three test concentrations of TRX. NSCs were exposed to TRX for 48 h followed by staining with Calcein AM as a live cell marker and propidium iodide as a dead cell marker. **A:** Represents the percentage of viable cells which was calculated from the total cell count. WST-1 was performed for differentiated culture of NSCs for 48 h exposed to 10µM Aß42. Test was performed in a 96 well plate. **B:** Represents the spectrophotometer absorbance values. Con; Control, TRX; troxerutin. Data are presented as mean±SEM. Experiments were performed as 5 replicates (n=5) (Supplementary, Page-2).

Following sentences have been added to the revised manuscript.

Oligomeric form of Aß42 is implicated in Alzheimer’s disease, a leading neurological disorder characterized by progressive loss of memory and cognitive functions (5, 6) (Results, Neuroprotective effect of TRX against Aß42 induced depression in NSCs differentiation, Page-16).

whilst natural products, such as flavonoids including TRX, have proven their efficacy against the Aß42 induced neurotoxicity both in *in vitro* and *in vivo* models (6-9). In this set of experiment, an *in vitro* model based upon NSCs isolated from SVZ of the developing mouse was established to unveil the neutralizing effect of TRX against the oligomeric Aß42 induced inhibition in the differentiation of NSCs and neurite growth. (Results, Neuroprotective effect of TRX against Aß42 induced depression in NSCs differentiation, Page-16).

the neurite growth in the differentiated neurons (2). Moreover, the literature reveals that lower concentrations of Aß42 exhibit neurogenerative effects rather than neurotoxicity through a compensatory mechanism of brain repair (3, 4). Finally, WST-1 cytotoxicity assay was performed to investigate the impact of Aß42 on the cell viability in differentiated culture for 48 h and a non-significant difference between the Control and the 1Aß42 (10 µM) was observed (SB Fig). (Results, Neuroprotective effect of TRX against Aß42 induced depression in NSCs differentiation, Page-16).

Moreover, short term exposure to Aß42 inhbits neurite growth of cultured PC12 cells due to an oxidative stress and mitochondrial dysfunction as described by researchers (2). (Discussion, page-23).

**(iv) Potential extracellular effects of your drug on Ab42 toxicity should be controlled.**

**Author’ response:** The flavonoids of different classes are capable of reducing the aggregation of Aß42. Rutin (6), toxifolin (7), theaflavin and gallo catechin gallate (8) are well known for their ability inhibit the formation of fibrils of Aß42 on extracellular reaction. Troxerutin flavonoid possesses all the structural requirements in its molecule prerequisite to its countertoxicity effects against the Aß42. Several effect based studies already revealed the neutralizing and neuroprotection effects of troxerutin against Aß42 induced spatial learning and memory defects in rat model from (9) partially by attenuating the synaptic toxicity in hippocampus cells as reported in an in vivo study (10), and neuroprotective effects against Aß42 neurotoxicities by ameliorating the functions of acetyl cholinesterase and those involved in hippocampus neural cells (11). The present study was an *in vitro* effect-based study to evaluate the possibility of considering troxerutin flavonoids against Amyloid-ß neurotoxicities using NSCs from the SVZ of the developing mammalian brain. Due to time and budget constraints we could not perform the studies to explore the extracellular chemical interactions between troxerutin flavonoid and the Aß42. Chemical interaction-based studies and molecular studies will be considered in our future projects.

**(v) all statements such as those about axons have to be supported by data.**

**Author’s replay:** Many of the supportive information regarding axonal growth was already present in the manuscript file. Please see the sentence enclosed in the brackets.

*“Another in vitro study reported that Aß42 on short term incubation significantly inhibited the axonal growth as well as synapsis formation in cultured cortical and hippocampus cells. These inhibitory effects of Aß42 are similar to those defined in transgenic mouse and Alzheimer pathology (**12**)”*

However, some additional supportive information about axonal growth have been added to the discussion portion of the revised manuscript as given below**.**

Prenylated flavonoid ENDF1 enhanced the axonal length and branching density in the cultured neurons from the dorsal ganglion by upregulating the expression of microtubule binding-protein gene DCX and maintaining the Ca^2+^ haemostasis in neurons (13). (13) (Discussion, page-22).

Oral administration of flavonoids rich dried root extracts of Chinese herb Scutellaria baicalensis  Georgi stimulated the axonal growth against experimentally induced spinal injury in a rat model by upregulating the expression of NF-H expression in neurons (14). Isoquercitrin flavonoids promoted the axonal elongation in of the cultured NG108-15 cells by reducing the activity of RhoA kinase (15) (Discussion, page-22).

**(vi) Effects on NSC differentiation should be shown (after all that is the ultimate proof of preservation of NSC stemness.**

**Author’s response:** We already performed the differentiation experiments (Differentiation experiment for TRX at 48 h, 7 days as well as differentiation experiment for TRX-Aß42 combination) for neuronal and astrocyte lineages and represented results in our manuscript upon 1^st^ submission. After extraction of NSCs from Mouse SVZ, we stained culture for NSCs stem cell markers to make sure that our cell pool chiefly consisted of NSCs. The same pool of cell was used for differentiation experiments. Please see the headings of differentiation experiments in the Results section of the manuscript file on 1^st^ submission. The main headings of these differentiation experiments are given in the brackets as follows

**[[***TRX enhanced neurite growth in neurons differentiated from NSCs; Neuroprotective effect of TRX against Aß42 induced depression in NSCs differentiation and* ***Fig****. 4A-D;* ***Fig****. 5A-D,* ***Fig****. 6,* ***Fig****. 7;* ***Fig****. 8A-D;* ***Fig****. 9;* ***Fig****, 10***]]**. (Results, Pages 11-16).

However, we added some additional Figs and description to the revised manuscript. These are given as below.

Fig 5. Effects of TRX on NNN after 7 days of differentiation. NSCs were differentiated for 7 days in the presence of TRX. Cells were fixed and immunostained for ß-tubulin III neuronal markers. Green cells represent neurons whilst blue rounded bodies indicate nuclei stained by nuclear dye DAPI. Control condition presented more neurons without neuritis (NNN) than the TRX as indicated by white arrows in the top left and top right images. Fluorescent images were captured with a 40 x objective of a microscope with scale bar measures 50 µm. (Results, TRX enhanced neurite growth in neurons differentiated from NSCs, Page-13)

Fig 6. Effects of TRX on NNN after 48 h of differentiation. NSCs were differentiated for 48 h in the presence of TRX. Cells were fixed and immunostained for ß-tubulin III neuronal markers and GFAP astrocyte marker. Green cells represent neurons, red cells indicate astrocytes and blue rounded bodies present nuclei stained by nuclear dye DAPI. Control condition exhibited more neurons without neuritis (NNN) than TRX as indicated by white arrows in the top left image and more cells co-stained for neuronal and astrocyte (yellow cells) indicated by a white arrow in the bottom left images. Fluorescent images were captured with a 40 x objective of a microscope with scale bar 50 µm. (Results, TRX enhanced neurite growth in neurons differentiated from NSCs, Page-13)

**Fig 9. Effect of TRX on neurite arborisation after 7 days of differentiation.** Neurite arborisation measurements were carried out for NSCs culture differentiated for 7 days with different concentrations of TRX. Around 100 neurons were included in the observation for each condition in each replicate. **A:** Represents the effects of TRX on neurite attachment point; AP and neurite ending points; EP. AP is an indicator of primary neurite number and EP is an indicator of neurite branching. **B:** Represents the neurite arborisation index which is described as the ratio of EP to that of AP. Data are presented as mean±SEM. *p≤0.05.

.(Results, TRX enhanced neurite growth in neurons differentiated from NSCs, Page-15).

Neurite arborisation was assessed by calculating arborisation index as reported in the literature (16). None of the tested concentrations of TRX affected the arborisation index after 7 days of differentiation. However, TRX significantly enhanced the number of neurite branching tips when compared to the Control (Fig 9) at both 50µM *(p=0.017)* and 100µM *(p=0.002)* concentrations. (Results,TRX enhanced neurite growth in neurons differentiated from NSCs, Page 14).

Effect of TRX on neurite arborisation index was non-significant at lower concentration but the higher concentrations of 50µM and 100µM significantly enhanced the number of neurite branching tips.(Discussion, Page-21).

**(vii) relevant literature should be cited.**

**Author’s replay:** The relevant literature has been added in multiple places of the result and discussion portions of the revised manuscript.

**Comments from Reviewer #1**

**1) -stemness" is determined by staining with nestin and GFAP. This is insufficient to examine the developmental state of the dispersed NSCs (cells). For example Sergent-Tanguayeyt al (2006) J Neurosci Res find that astroglial cells can be nestin+/GFAP+. Additional studies should include more rigorous characterization of the cells used in the work. This is critical information needed to interprete all other studies.**

**Author’ response:** We are thankful to reviewer for his comments. In this study, we wanted to use a simple readout that allows identifying the amount of reactive glia, which is to be analysed by a co-staining of nestin and GFAP as an appropriate tool. We did not want to perform a deep analysis of all neural stem cell marker. There are many, and many with individual aspects. Nestin and GFAP seemed to us the most appropriate for the characterisation of reactive glia.

Immunostaining is a widely and commonly used technique for the characterization of NSCs. Nestin, GFAP, TLX, DCX, SOX2 and Atohl are the markers used in common practice for the identification of NSCs/Neural progenitor cells (NPCs) in *in vitro* culture . DCX is the marker for NPCs but not for pure NSCs. Atoh1 is the marker for NSCs of the cochlear nucleus. SOX2 is the marker for the identification of NSCs during earlier phase of the development of nervous system. Nestin is the most widely used marker for NSCs from both developing and adult nervous system. Nestin is expressed by both NSCs and Neural progenitor cells. There is a category of NSCs which are immunoreactive to glial marker in addition to the nestin. These cells possess radial glial like morphology and are purely NSCs. Such a population of cells which express both nestin and GFAP markers occurs specifically in the SVZ of the brain and are NSCs and should not be interpreted as neural progenitor (17-19). These are reasons for which we selected nestin and GFAP markers for the early identification of our NSCs isolated from the SVZ of the postnatal mouse.

Following paragraph has been added to the revised manuscript.

Routinely, NSCs are identified in an *in vitro* culture by the immunostaining of NSCs/ neural progenitor cells (NPCs) markers namely DCX, Atoh1, SOX2, Nestin and GFAP. DCX is a marker for NPCs and not pure NSCs. Atoh1 is the marker for NSCs of the Cochlar nucleus. SOX2 is the marker of NSCs in the developing brain during early embryonic phase. Nestin is the widely employed marker for both NSCs and NPCs in the developing and adult nervous system (17-19). (Discussion, page-19).

**2) The neurosphere data in Fig. 3 is unconvincing. Only a single neurosphere is shown (and in many cases these images are out of focus). A representative field including many spheres should be shown.**

**Author response:** We are thankful to the reviewer comments and advice and apology for the bad quality. Fig. 3 has been replaced by a new picture with many neurospheres in a microscopic field.

Fig 3. Neurosphere clonogenic assay for NSCs proliferation. The medium of Control+ve was supplemented with EGF and FGF growth factors. Growth factors were completely omitted from the Control-ve and all TRX treatments. Cells proliferated rapidly in positive control and formed neurospheres whose size increased with the increase in the incubation period. Cells in all other treatment conditions failed to proliferate and completely died on 7^th^ day of observation. Pictures were captured with a 10 x objective of a phase-contrast microscope. (Results, TRX effects on proliferation, page-11).

**3) (A)-For Fig. 4, it does not appear that the impact of TRX on NSC differentiation is ever examined. Cultures would need to be temporally characterized for the presence of NSC, immature/mature neurons, oligodendrocytes, and glial numbers - this should be done and the percentage of each cell type included in the analysis. (B)-It is also unclear what NNN cells are - could these be dead or dying neurons? (C)-Other cell types? Images are needed to support these studies.**

**A)-For Fig. 4, it does not appear that the impact of TRX on NSC differentiation is ever examined. Cultures would need to be temporally characterized for the presence of NSC, immature/mature neurons, oligodendrocytes, and glial numbers - this should be done and the percentage of each cell type included in the analysis**

**Author’s response:** Differentiation experiments were carried out for TRX alone as well as in combination with Aß42. Neurons were identified by a neuronal marker ß-tubulin III and astrocytes were identified by GFAP glial cell marker. Not only the percentage of differentiated neurons and astrocytes were calculated but their morphological parameters were studied in detail. All these results were already given in the result portion and complete discussion with relevant literature supports were given in the Discussion portion of our manuscript upon 1^st^ submission. Please see the following headings in our first submitted manuscript file.

*Result: “TRX enhanced neurite growth in neurons differentiated from NSCs”*(Results, Page 11-15 in manuscript 1^st^ submission).

*“****Fig 4.*** *TRX effect on NSCs differentiation after 7 days and 48 h incubation....”* (Results, Page-12 in manuscript 1^st^ submission).

***“Fig 5.*** *Effects of TRX on morphological parameters of neurons and astrocytes differentiated from NSCs after 7 days and 48 h of incubation.....”* (Results, Page-13 in manuscript 1^st^ submission).

*“Fig 6. Effect of TRX on neurite growth of neurons and soma area of astrocyte after 7 days of incubation”.* (Results, Page-13 in manuscript 1^st^ submission)

*“Fig 7.Effect of TRX on neurite growth of neurons and soma area of astrocyte after 48 h of incubation”.* (Results, Page-14 in manuscript 1^st^ submission).

Results*. “Neuroprotective effects of TRX against Aß42 induced depression in NSCs differentiation”* (Results, Page-14-16 in manuscript 1^st^ submission).

*“Fig 8.Neuroprotective effects of TRX flavonoid against Aß42 induced depression of differentiation after 48 h of differentiation on ECM coated glass coverslips.........”*(Results, Page-15 in manuscript 1^st^ submission).

*“Fig 9. Neuroprotective effect of TRX against Aß induced inhibition in amount of neurons and astrocytes after 48 h of differentiation......”*(Results, Page-15 in manuscript 1^st^ submission).

*“Fig 10. Neuroprotective effect of TRX against Aß induced neurite growth inhibition after 48 h incubation......”*(Results, Page-16 in manuscript 1^st^ submission).

Discussion for Differentiation experiment. (Discussion, Pages19-21 in manuscript 1^st^ submission).

For differentiation, assay we include the two most important lineages of NSCs (neurons and astrocytes) in our observations and did not include oligodendrocytes. The reason for not including oligodendrocytes in our study was that we found most of the screening studies focussed either on the neuronal cell culture system (20) or neuronal/astrocyte co-culture. Neurons are the basic physiological unit of the nervous system and are highly vulnerable to the chemical stimuli. Astrocytes are the second most important cells of the nervous system since they regulate the metabolic activity of the neurons and also promote neuronal tolerance to the toxic stimuli. Additionally, astrocytes promote neuronal migration and synaptic activity. Astrocyte response to the extracellular chemical stimuli very quickly and any perturbation in astrocyte functions ultimately leads to the strong impact on the neurons so neuronal/astrocyte co-culture is a very relevant in-vitro model for screening neuromodulating compounds (21-24). Oligodendrocytes constitute only 5-8% of the total glial cells and play a passive role in the nervous system (formation of myelin sheath) and earned little attention of the researchers for screening compounds (23, 24). In the last a very complex culture system that simulates the myelin-sheath effect of Oligodendrocytes is required for screening the effect of exogenous substances on oligodendrocytes. Here we wanted to introduce a relatively simple approach that indeed has options to be expanded. We found only limited studies reporting the application oligodendrocyte cell culture for screening selective compounds (25, 26). Since the present study is an attempt to establish a *in vitro assay* system based upon neural stem cells from the SVZ of a postnatal mouse to explore the neurogenerative and the neuroprotective effects of flavonoid TRX against the Aß42 induced neurotoxicity so we tried to adhere with those neural cell culture conditions which have been used extensively by the researchers for screening environmental toxicants and have been frequently published.

We added some new Figures as well as new information concerning differentiation experiments in the result and discussion portions of the revised manuscript. Figures numbers have been rearranged in the revised manuscript.

Fig 5. Effects of TRX on NNN after 7 days of differentiation. NSCs were differentiated for 7 days in the presence of TRX. Cells were fixed and immunostained for ß-tubulin III neuronal markers. Green cells represent neurons whilst blue rounded bodies indicate nuclei stained by nuclear dye DAPI. Control condition presented more neurons without neuritis (NNN) than the TRX as indicated by white arrows in the top left and top right images. Fluorescent images were captured with a 40 x objective of a microscope with scale bar measures 50 µm. (Results, TRX enhanced neurite growth in neurons differentiated from NSCs, Page-13).

Fig 6. Effects of TRX on NNN after 48 h of differentiation. NSCs were differentiated for 48 h in the presence of TRX. Cells were fixed and immunostained for ß-tubulin III neuronal markers and GFAP astrocyte marker. Green cells represent neurons, red cells indicate astrocytes and blue rounded bodies present nuclei stained by nuclear dye DAPI. Control condition exhibited more neurons without neuritis (NNN) than TRX as indicated by white arrows in the top left image and more cells co-stained for neuronal and astrocyte (yellow cells) indicated by a white arrow in the bottom left images. Fluorescent images were captured with a 40 x objective of a microscope with scale bar 50 µm. (Results, TRX enhanced neurite growth in neurons differentiated from NSCs, Page-13)

**Fig 9. Effect of TRX on neurite arborisation after 7 days of differentiation.** Neurite arborisation measurements were carried out for NSCs culture differentiated for 7 days with different concentrations of TRX. Around 100 neurons were included in the observation for each condition in each replicate. **A:** Represents the effects of TRX on neurite attachment point; AP and neurite ending points; EP. AP is an indicator of primary neurite number and EP is an indicator of neurite branching. **B:** Represents the neurite arborisation index which is described as the ratio of EP to that of AP. Data are presented as mean±SEM. *p≤0.05.

.(Results, TRX enhanced neurite growth in neurons differentiated from NSCs, Page-15).

Neurite arborisation was assessed by calculating arborisation index as reported in the literature (16). None of the tested concentrations of TRX affected the arborisation index after 7 days of differentiation. However, TRX significantly enhanced the number of neurite branching tips when compared to the Control (Fig. 9) at both 50µM *(p=0.017)* and 100µM *(p=0.002)* concentrations..(Result,TRX enhanced neurite growth in neurons differentiated from NSCs, Page.14).

whilst natural products, such as flavonoids including TRX, have proven their efficacy against the Aß42 induced neurotoxicity both in *in vitro* and *in vivo* models (6-9). In this set of experiment, an *in vitro* model based upon NSCs isolated from SVZ of the developing mouse was established to unveil the neutralizing effect of TRX against the oligomeric Aß42 induced inhibition in the differentiation of NSCs and neurite growth. (Result,Neuroprotective effect of TRX against Aß42 induced depression in NSCs differentiation, Page. 16).

the neurite growth in the differentiated neurons (2). Moreover, the literature reveals that lower concentrations of Aß42 exhibit neurogenerative effects rather than neurotoxicity through a compensatory mechanism of brain repair (3, 4). Finally, WST-1 cytotoxicity assay was performed to investigate the impact of Aß42 on the cell viability in differentiated culture for 48 h and a non-significant difference between the Control and the 1Aß42 (10 µM) was observed (Fig SB). (Result,Neuroprotective effect of TRX against Aß42 induced depression in NSCs differentiation, Page. 16).

Effect of TRX on neurite arborisation index was non-significant at lower concentration but the higher concentrations of 50µM and 100µM significantly enhanced the number of neurite branching tips.(Discussion, Page-21).

**(B)-It is also unclear what NNN cells are - could these be dead or dying neurons?**

**Author’s response:** We thank the reviewer for this question and see his or her concern. But we can provide data that easily show the living character of these cells. We performed live-dead assay for different concentrations of TRX and found that all concentration used for differentiation experiment were found safe with no signs of toxicity (Fig SA). NNN cells are included in this culture. We see in the NNN population a group of cells that is not sufficiently triggered to develop neurites but is nevertheless well alive. The NNN population gives a very good impression upon the affectivity of compounds to increase neurite outgrowth. When neuronal cells are not stimulated by i.e. growth factors, only a small part of the cells does provide neurites (27). Our WST-1 assay showed that concentration of Aß42 we used in our TRX counter toxicity differentiation experiment non-significant affected the viability of cells when compared to the control (Fig SB) so the possibility that NNN were dead cells is excluded.

Following informations were added to the supplementary file.

**Fig S. Live-dead and WST-1 cytotoxicity assays.** Calcein/propidium iodide live-dead assay was performed for three test concentrations of TRX. NSCs were exposed to TRX for 48 h followed by staining with Calcein AM as a live cell marker and propidium iodide as a dead cell marker. **A:** Represents the percentage of viable cells which was calculated from the total cell count. WST-1 was performed for differentiated culture of NSCs for 48 h exposed to 10µM Aß42. Test was performed in a 96 well plate. **B:** Represents the spectrophotometer absorbance values. Con; Control, TRX; troxerutin. Data are presented as mean±SEM. Experiments were performed as 5 replicates (n=5) (Supplementary, Page-2).

Moreover, a non-significant difference concerning NSCs viability was also observed between the Control and 100µM TRX (Fig SA)**.** (Results, TRX enhanced neurite growth in neurons differentiated from NSCs, page-14).

the neurite growth in the differentiated neurons (2). Moreover, the literature reveals that lower concentrations of Aß42 exhibit neurogenerative effects rather than neurotoxicity through a compensatory mechanism of brain repair (3, 4). Finally, WST-1 cytotoxicity assay was performed to investigate the impact of Aß42 on the cell viability in differentiated culture for 48 h and a non-significant difference between the Control and the 1Aß42 (10 µM) was observed (Fig SB). (Results, TRX enhanced neurite growth in neurons differentiated from NSCs, page-16).

**(C) Other cell types? Images are needed to support these studies.**

**Author’s response:** Term “other cells” was used for those which were neither immunoreactive to neuronal marker ß-tubulin III nor astrocytes marker GFAP. These were simply calculated from the phase contrast and DAPI nuclei merge pictures. Definitely, these cells are expressing specific markers, which have not been in the focus of our study, as mentioned above. In the Fig 12 as given below, white arrows indicate the nuclei without soma as these were the cells belonged to a category the markers of which were not included in our study. Such cells were termed as “other cells”

Fig 12. Neuroprotective effect of TRX against Aß42 induced inhibition in the amount of neurons and astrocytes after 48 h of differentiation. Green cells present ß-tubulin III neurons which were found denser in control and Aß42+TRX. Red cells represent GFAP astrocytes which were observed in both leaf-like and star-like morphology. Astrocytes were relatively denser in Control as compared to other treatment conditions. Pictures were captured with a 40 x objective of a fluorescent microscope. Scale bar is 50 µm. (Results, Neuroprotective effect of TRX against Aß42induced depression in NSCs differentiation Page-17).

**5). (A)-It is unclear why AB42 was used to explore TRX-mediated survival. (B)- Is it known where TRX exposure alters AB42 oligmerization/ability to form fibrils? (C)-Could what is considered cell survival be a reduction in AB42 in vitro toxicity? (D)- Does TRX treatment cause pro-survival molecular changes (described in paper below)? (E)-Are the GFAP+/nestin+ cell simply astrocytes. (F)-Finally, astrocytes play a role in amyloid production - might differences in astrocye number impact these studies? (G)-This study mirrors a published study: Farajdokht et al. 2017 PMID 29285004. This work should be acknowledged and included in the Discussion.**

**(A)-It is unclear why AB42 was used to explore TRX-mediated survival?**

**Authors response:** Researcher reported that aggregated form of Aß (42) is implicated in neurological disorders such as Alzheimer’s. Oxidative stress plays a vital role in the deposition, accumulation and aggregation of Amyloid ß peptides. Under oxidative stress overproduction of reactive oxygen species leads to a mutation in genes regulating the production and proper folding of Amyloid-ß into the functional peptide. Antioxidants such as rutin flavonoids have proven their efficacy against neurological disorders mediated by the aggregation of Aß (6). Troxerutin is a water-soluble derivative of rutin flavonoid also occurs naturally. Troxerutin showed neutralizing and neuroprotection against Aß (42) induced spatial learning and memory defects in rat model from (9) partially by attenuating the synaptic toxicity in hippocampus cells as reported in an in vivo study (10). Troxerutin also showed its neuroprotective effects against Aß (42) neurotoxicities by ameliorating the functions of acetyl cholinesterase and those involved in hippocampus neural cells (11). As amyloid-ß aggregation is a hallmark of leading neurological disorder Alzheimer’s and plant flavonoids have proven their efficacy against Amyloid-ß neurotoxicity so we used troxerutin to explore if it shows neuroprotective effects aginst Aß (42) induced toxicity on the morphology of neurons and astrocytes during early differentiation of NSCs isolated from SVZ of the postnatal mouse.

Following sentences have been added to the revised manuscript.

Oligomeric form of Aß42 is implicated in Alzheimer’s disease, a leading neurological disorder characterized by progressive loss of memory and cognitive functions (5, 6).. (Results, Neuroprotective effect of TRX against Aß42 induced depression in NSCs differentiation,Page. 16).

whilst natural products, such as flavonoids including TRX, have proven their efficacy against the Aß42 induced neurotoxicity both in *in vitro* and *in vivo* models (6-9). In this set of experiment, an *in vitro* model based upon NSCs isolated from SVZ of the developing mouse was established to unveil the neutralizing effect of TRX against the oligomeric Aß42 induced inhibition in the differentiation of NSCs and neurite growth**.** (Results,Neuroprotective effect of TRX against Aß42 induced depression in NSCs differentiation,Page. 16).

**(B)-Is it known where TRX exposure alters AB42 oligmerization/ability to form fibrils?**

**Authors Response:** The fact that flavonoids of different classes are capable of reducing the aggregation of Aß (42). Rutin (6), toxifolin (7), theaflavin and gallo catechin gallate (8) are well known for their ability to inhibit the fibrils formation of Aß42 on extracellular reaction. Although we found effect based studies about the inhibitory effects of troxerutin flavonoid against Aß 42 neurotoxicity (10, 11) but data concerning the extracellular activity of troxerutin to prevent fibrils formation of Aß 42 is lacking. The ability of flavonoids to inhibit the fibril formation of Aß 42 largely depends upon the number of aromatic rings and the number of hydroxyl functional groups present in the flavonoids molecule. Greater is the number of these functional groups strong is their inhibitory effects against Aß42 fibrils formations. The aromatic ring structure of flavonoids form covalent bonds with the hydrophobic amino acids residues and hydroxyl groups form hydrogen bonds with hydrophilic amino acid residues of Aß 42 peptide backbone and ultimate result is the prevention of fibrils formation. Gallocatechingallate and theaflavin flavonoids showed 100% efficacy in preventing fibrils formation of Aß 42 in an extracellular chemical reaction because of the abundance in aromatic ring and hydroxyl groups in their structures as compared to other compounds tested (8). Following **Table 1** shows the number of aromatic rings and hydroxyl groups in flavonoids of anti-Aß42 activities. Table shows that troxerutin stands on 2^nd^ position concerning the functional groups implicated in preventing the fibrillization of Aß42. However, additional studies are required to prove this fact. Because of our time limitations and budget constraints of this project, we could not perform this kind of studies but these of course are in our priority for future projects. Moreover, the present study was an effect based study in which our main goal was to assure if our *in vitro* model based upon NSCs from the developing mouse could successfully explore the biological effects of Aß 42 and Troxerutin.

**Table 1.**

| **Flavonoids** | **No. of aromatic rings** | **No. of hydroxyl ring** | **References** |
| --- | --- | --- | --- |
| Theaflavin | 4 | 7 | (28) |
| Gallocatechingallate | 3 | 8 | (29) |
| Rutin | 3 | 10 | (30) |
| Troxerutin | 3 | 11 | (31) |

Folowing sentences have been added to the revised manuscript file.

Countertoxicity effects of a flavonoid against Aß 42 depend upon the ability of a flavonoid to prevent the fibrillization of the later (6). Two major structural requirements for the anti-fibrilization effect of a flavonoid molecule involve the number of aromatic rings and the number of hydroxyl groups present in the molecule. Aromatic rings bind with the hydrophobic amino acid residues through covalent bonds and hydroxyl groups of a flavonoid interact with hydrophilic amino acid residues of the peptide backbone of Aß 42 leading to the disaggregation effects or prevention of fibrillization. The number of these functional groups are, therefore, directly proportional to the antifibrils activity against Aß 42. Gallocatechin gallate and theaflavin exhibited 100% efficacy in preventing fibrils formation of Aß 42 in an extracellular chemical reaction since these molecules provide a higher number of aromatic rings and hydroxyl groups in their structures as compared to other compounds tested (Phan 2019). A number of these aromatic rings and reactive hydroxyl groups in TRX molecule (31) is comparable to that of theaflavin (28), rutin (30) and more than that of gallocatechin gallate (29) so it was assumed that the neutralizing activity of TRX against Aß 42 induced inhibition in neuronal differentiation and neurite growth in our experiments might be due to the disaggregating or antifibrillization effects of TRX. Future detailed studies are needed to prove if TRX prevents fibrillization of Aß42 in a reaction mixture. (Discussion, Page,24).

**(C)-Are the GFAP+/nestin+ cell simply astrocytes?**

**Authors response;** Cells which express both nestin and GFAP markers have radial glial morphology are purely NSCs occur specifically in the SVZ of the brain and should not be interpreted as neural progenitor (17-19). This information was already given in the manuscript file upon 1^st^ Submission.

Please the paragraph in the following paragraph.

*There exists a class of NSCs which co-express nestin and the reactive glial cell marker GFAP. So both Nestin and GFAP are considered as NSCs markers (**32**). NSCs which co-express Nestin and GFAP are radial glial cells which have neural stem cell properties (**33**,* *34**). These cells predominantly exist in the SVZ of both developing and adult brain and are capable of differentiation into neurons, oligodendrocytes and astrocytes in vitro (**35**,* *36**). We used both nestin and GFAP immunostaining to identify NSCs from our SVZ culture proliferated for 7 days in the presence of different concentrations of TRX* (Discussion, page 18, Manuscript file 1^st^ Submission).

**(D)-Is it known where TRX exposure alters AB42 oligmerization/ability to form fibrils?**

**Authors response:** Data regarding the direct effect of troxerutin on Aß 42 aggregation is lacking. Literature reported that rutin flavonoids having structural similarity to the TRX flavonoid, reduced the neurotoxicity and memory defects in the mouse by decreasing the aggregation of Aß42. For future studies, it will be very useful if inhibitory effects of TRX on Aß42 are explored in an extracellular setup. In present our major intension was to develop and use primary NSCs based *in vitro* model for early screening of biological impacts Aß 42 and TRX. Due to time and budget limitations, we could not perform the extracellular study in the present project but we will consider this for future projects.

**(E)-Could what is considered cell survival be a reduction in AB42 in vitro toxicity?**

**Authors response:** The concentration of Aß (42) we use in our study was that reported by researchers for its inhibitory effects on %age of neurons and astrocytes differentiated from cultured hippocampus NSCs culture (4). In a separate experiment, we evaluated the effect of 10 µM Aß 42 on the viability of neurons/astrocyte differentiated culture for 48 h by WST-1 viability assay and found that Aß42 insignificantly affected the viability of cells when compared to the vehicle control **(Fig. SB)** Researchers reported that aggregated form of Aß 42 inhibited the neurite outgrowth of cultured cortical neurons at a higher dose by decreasing the adhesion of cells to the extracellular matrix. Neuronal adhesion to the extracellular matrix is prerequisite for neurite outgrowth and cell migration. In vivo data also suggest that Aß42 disrupted the cell adhesion mechanism (37). The other possible mechanism of the neuroinhibitory effects of the Aß42 is that at this concentration it decreased the neuronal and astrocyte differentiation of NSCs by accelerating senescence mechanism (4).

Following Fig along with description have been added to the revised manuscript.

Moreover, short term exposure to Aß42 inhbits neurite growth of cultured PC12 cells due to an oxidative stress and mitochondrial dysfunction as described by researchers (2). (Discussion, Page-23).

**Fig S. Live-dead and WST-1 cytotoxicity assays.** Calcein/propidium iodide live-dead assay was performed for three test concentrations of TRX. NSCs were exposed to TRX for 48 h followed by staining with Calcein AM as a live cell marker and propidium iodide as a dead cell marker. **A:** Represents the percentage of viable cells which was calculated from the total cell count. WST-1 was performed for differentiated culture of NSCs for 48 h exposed to 10µM Aß42. Test was performed in a 96 well plate. **B:** Represents the spectrophotometer absorbance values. Con; Control, TRX; troxerutin. Data are presented as mean±SEM. Experiments were performed as 5 replicates (n=5) (Supplementary, Page-2).

**(F)-Finally, astrocytes play a role in amyloid production - might differences in astrocye number impact these studies?**

**Author response:** Environmental toxicants or proinflammatory stimuli trigger the proliferation and activation of immunoreactive astrocytes whose basic role is to destroy the noxious stimuli but they also contribute in the progression of the disease by synthesizing Aß peptides precursors and accumulation and spreading of neurotoxic oligomeric Aß in the brain which leads to neuronal toxicities (38, 39). Plant flavonoids because of their antioxidant and anti-inflammatory role, are found useful in astrocyte mediated Aßneurotoxiity, for, instance, curcumin promoted the neuronal cell survival against the against Aß 42 neurotoxicity by attenuating the inflammatory response of astrocytes through the inhibition of NF-κB pathway in the neuronal/astrocytes mixed culture from APP/S1 transgenic mouse (40). How over, in our study the difference between TRX and Aß42 concerning effects on astrocytes %age and astrocytes soma area were insignificant so this phenomenon might not play a role in the neuroprotective effects of TRX against Aß 42 neurotoxicity. Molecular studies are required to unveil the role of astrocytes in Aß42 toxicity.

**(G)-This study mirrors a published study: Farajdokht et al. 2017 PMID 29285004. This work should be acknowledged and included in the Discussion?**

**Authors response:** The above study has already been acknowledged in the discussion please see **“**An *in vivo* study revealed that TRX protected hippocampus neurons from the neurotoxic effects of Aß42 by ameliorating antioxidant enzymes and attenuating elevated acetylcholinesterase enzyme levels” (Discussion, Page 20-21 (11) .

Following information has been added to the revised manuscript

Additionally TRX also reduced the apoptosis on chronic treatment of 14 days (11). (11)(Discussion, Page -23).

**Overall:
The image quality is very poor for the majority of figures, making the review significantly more difficult. The manuscript would be improved by careful review and editing my a native English speaker.**

**Author’ response:** Quality of the all figures in the revised manuscript has been improved. The whole manuscript was thoroughly reviewed and all grammar mistakes such as spelling error, misuse of capital words, spacing issues, abbreviation misuse and other have been corrected in the revised manuscript by a native English speaking person and using Grammarly/ Ginger software.

**Reviewer #2:**

**Overall the paper is easily read, but there are still some minor problems in language and spelling.**

**A through out checking is recommended. A previous study has shown that behaviour of stem cells depends upon the physiochemical properties of the material and the drugs.**( <https://doi.org/10.1002/admi.201900572>). **Please discuss your article in keeping with the article and explain which property of your troxerutin flavonoid resulted in differentiation of Cells?**

**Author’s response:** The whole manuscript was thoroughly reviewed and all grammar mistakes such as spelling error, misuse of capital words, spacing issues, abbreviation misuse and other have been corrected in the revised manuscript by a native English speaking person and using Grammarly/ Ginger software.

The Review article linked shared by the reviewer is based upon the pharmaceutical formulation techniques to enhance the efficacy of bioactive compounds by polymers and crossed linked polymers. Since our study was an effect based study of pure TRX on NSCs fate and less likely to address the physical and chemical optimization of TRX formulation.

Reduce oxidative stress through antioxidant compounds increases the neuronal differentiation of NSCs from mammalians (41).Troxerutin possesses antioxidant properties by reacting with reactive oxygen species (42). Neuroprotective and neurogenerative effects of TRX through its anti-oxidative actions in the brain tissues of the rat were reported by researchers in their study (43). Keeping in views these facts, we assume that anti-oxidative actions of TRX might play a pivotal role in enhancing neuronal differentiation and neurite growth in our culture. However, because the present study was a basic effect based study of TRX and due to time and budget constraints, we could perform anti-oxidative and molecular mechanism studies. Detailed anti-oxidation studies with molecular mechanism evaluation will be the main part of our future project.

Following paragraph have been added to the revised manuscript

Reduced oxidative stress stimulates the neuronal differentiation of NSCs (41). TRX exhibits antioxidant activities by interacting with reactive oxygen species (42). Since TRX exerts neuroprotective and neurogenerative effects through its anti-oxidative actions in the brain tissues of rat (43), it was assumed that anti-oxidation activity might also be associated with enhanced neuronal differentiation and neurite growth. Detailed anti-oxidation and molecular studies are required to prove this assumption (Discussion, Page-22-23).

**References**

1.	Papuć E, Rejdak K. Does myelin play the leading role in Alzheimer’s disease pathology. J Alzheimers Dis Parkinsonism. 2017;7(321):2161-0460.1000321. doi: 10.4172/2161-0460.1000321.

2.	Kurz C, Ungerer I, Lipka U, Kirr S, Schütt T, Eckert A, et al. The metabolic enhancer piracetam ameliorates the impairment of mitochondrial function and neurite outgrowth induced by ß‐amyloid peptide. Br J Pharmacol. 2010;160(2):246-57. doi: 10.1111/j.1476-5381.2010.00656.x. PMC2874847.

3.	Brothers HM, Gosztyla ML, Robinson SR. The physiological roles of amyloid-β peptide hint at new ways to treat Alzheimer's disease. Front Aging Neurosci. 2018;10:118. doi: 10.3389/fnagi.2018.00118. PMC5996906.

4.	He N, Jin W, Lok K, Wang Y, Yin M, Wang Z. Amyloid-β 1–42 oligomer accelerates senescence in adult hippocampal neural stem/progenitor cells via formylpeptide receptor 2. Cell Death Dis. 2013;4(11):e924. doi: 10.1038/cddis.2013.437. PMC3847315.

5.	Irwin K, Sexton C, Daniel T, Lawlor B, Naci L. Healthy Aging and Dementia: Two Roads Diverging in Midlife? Front Aging Neurosci. 2018;10. doi: 10.3389/fnagi.2018.00275. PMC6156266.

6.	Zuo L, Hemmelgarn BT, Chuang C-C, Best TM. The role of oxidative stress-induced epigenetic alterations in amyloid-β production in Alzheimer’s disease. Oxid Med Cell Longev. 2015;2015. doi: 10.1155/2015/604658. PMC4620382.

7.	Sato M, Murakami K, Uno M, Nakagawa Y, Katayama S, Akagi K-i, et al. Site-specific inhibitory mechanism for amyloid β42 aggregation by catechol-type flavonoids targeting the Lys residues. J Biol Chem. 2013;288(32):23212-24. doi: 10.1074/jbc.M113.464222. PMC3743493.

8.	Phan HT, Samarat K, Takamura Y, Azo-Oussou AF, Nakazono Y, Vestergaard MdC. Polyphenols Modulate Alzheimer’s Amyloid Beta Aggregation in a Structure-Dependent Manner. Nutrients. 2019;11(4):756. doi: 10.3390/nu11040756. PMC6521010.

9.	Babri S, Amani M, Mohaddes G, Alihemmati A, Ebrahimi H. Protective effects of troxerutin on β-amyloid (1-42)-induced impairments of spatial learning and memory in rats. Neurophysiology. 2012;44(5):387-93. doi: 10.1007/s11062-012-9309-6.

10.	Babri S, Mohaddes G, Feizi I, Mohammadnia A, Niapour A, Alihemmati A, et al. Effect of troxerutin on synaptic plasticity of hippocampal dentate gyrus neurons in a β-amyloid model of Alzheimer׳ s disease: An electrophysiological study. Eur J Pharmacol. 2014;732:19-25. doi: 10.1016/j.ejphar.2014.03.018.

11.	Farajdokht F, Amani M, Bavil FM, Alihemmati A, Mohaddes G, Babri S. Troxerutin protects hippocampal neurons against amyloid beta-induced oxidative stress and apoptosis. EXCLI journal. 2017;16:1081. doi: 10.17179/excli2017-526. PMC5735350.

12.	Evans NA, Facci L, Owen DE, Soden PE, Burbidge SA, Prinjha RK, et al. Aβ1–42 reduces synapse number and inhibits neurite outgrowth in primary cortical and hippocampal neurons: A quantitative analysis. J Neurosci Methods. 2008;175(1):96-103. doi: 10.1016/j.jneumeth.2008.08.001. 18761375.

13.	Bieler L, Vogl M, Kirchinger M, Urmann C, Riepl H, Bandtlow CE, et al. The prenylflavonoid ENDF1 overrules central nervous system growth inhibitors and facilitates regeneration of DRG neurons. Front Cell Neurosci. 2019;13:332. doi: 10.3389/fncel.2019.00332.

14.	Zhang Q, Zhang L-X, An J, Yan L, Liu C-C, Zhao J-J, et al. Huangqin flavonoid extraction for spinal cord injury in a rat model. Neural regeneration research. 2018;13(12):2200. doi: 10.4103/1673-5374.241472. PMC6199921.

15.	Palazzolo G, Horvath P, Zenobi-Wong M. The flavonoid isoquercitrin promotes neurite elongation by reducing RhoA activity. PLoS One. 2012;7(11):e49979. doi: 10.1371/journal.pone.0049979. Pmc3510166.

16.	Ho S-Y, Chao C-Y, Huang H-L, Chiu T-W, Charoenkwan P, Hwang E. NeurphologyJ: an automatic neuronal morphology quantification method and its application in pharmacological discovery. BMC Bioinformatics. 2011;12(1):230. doi: 10.1186/1471-2105-12-230. PMC3121649.

17.	Bazan E, Alonso F, Redondo C, Lopez-Toledano M, Alfaro JM, Reimers D, et al. In vitro and in vivo characterization of neural stem cells. Histol Histopathol. 2004. doi: 10.14670/HH-19.1261.

18.	Zhang J, Jiao J. Molecular biomarkers for embryonic and adult neural stem cell and neurogenesis. BioMed research international. 2015;2015. doi: 10.1155/2015/727542. PMC4569757.

19.	Völker J, Engert J, Völker C, Bieniussa L, Schendzielorz P, Hagen R, et al. Isolation and Characterization of Neural Stem Cells from the Rat Inferior Colliculus. Stem cells international. 2019;2019. doi: 10.1155/2019/5831240. PMC6875198.

20.	Coullery RP, Ferrari ME, Rosso SB. Neuronal development and axon growth are altered by glyphosate through a WNT non-canonical signaling pathway. Neurotoxicology. 2016;52:150-61. doi: 10.1016/j.neuro.2015.12.004. 26688330.

21.	Anderl JL, Redpath S, Ball AJ. A neuronal and astrocyte co-culture assay for high content analysis of neurotoxicity. JoVE (Journal of Visualized Experiments). 2009(27):e1173. doi:

22.	De Simone U, Caloni F, Gribaldo L, Coccini T. Human co-culture model of neurons and astrocytes to test acute cytotoxicity of neurotoxic compounds. Int J Toxicol. 2017;36(6):463-77. doi:

23.	Jiang D, Lim S, Kwak M, Ryu YK, Mintz CD. The changes of oligodendrocytes induced by anesthesia during brain development. Neural regeneration research. 2015;10(9):1386. doi:

24.	Deng W, Poretz RD. Oligodendroglia in developmental neurotoxicity. Neurotoxicology. 2003;24(2):161-78. doi:

25.	O'Sullivan A, Lange S, Rotheneichner P, Aigner L, Rivera FJ, Couillard-Despres S. Dimethylsulfoxide inhibits oligodendrocyte fate choice of adult neural stem and progenitor cells. Front Neurosci. 2019;13:1242. doi:

26.	Bénardais K, Kotsiari A, Škuljec J, Koutsoudaki PN, Gudi V, Singh V, et al. Cuprizone [bis (cyclohexylidenehydrazide)] is selectively toxic for mature oligodendrocytes. Neurotox Res. 2013;24(2):244-50. doi:

27.	Schafer K-H, Mestres P, Marz P, Rose-John S. The IL-6/sIL-6R fusion protein hyper-IL-6 promotes neurite outgrowth and neuron survival in cultured enteric neurons. J Interferon Cytokine Res. 1999;19(5):527-32. doi: 10.1089/107999099313974.

28.	Leung LK, Su Y, Chen R, Zhang Z, Huang Y, Chen Z-Y. Theaflavins in black tea and catechins in green tea are equally effective antioxidants. The Journal of nutrition. 2001;131(9):2248-51. doi: 10.1093/jn/131.9.2248.

29.	Wilkins C, De Bruijn J, Korver O, Frost D, Weinges K. Isolation and identification of (—)‐gallocatechin gallate and circular dichroism of green tea flavanols. J Sci Food Agric. 1971;22(9):480-4. doi: 10.1002/jsfa.2740220913.

30.	Enogieru AB, Haylett W, Hiss DC, Bardien S, Ekpo OE. Rutin as a potent antioxidant: implications for neurodegenerative disorders. Oxid Med Cell Longev. 2018;2018. doi: 10.1155/2018/6241017. PMC6040293.

31.	Xin X, Zhang M, Li X, Lai F, Zhao G. Biocatalytic synthesis of acylated derivatives of troxerutin: their bioavailability and antioxidant properties in vitro. Microbial cell factories. 2018;17(1):130. doi: 10.1186/s12934-018-0976-x. PMC6106897.

32.	Zalfa C, Grasselli C, Santilli G, Ferrari D, Lamorte G. GFAP Delta as Divergent Marker of Human Glial Progenitors. Stem Cell Res Ther. 2018;8(437):2. doi: 10.4172/2157-7633.1000437.

33.	Berg DA, Bond AM, Ming G-l, Song H. Radial glial cells in the adult dentate gyrus: what are they and where do they come from? F1000Research. 2018;7. doi: 10.12688/f1000research.12684.1. PMC5840617.

34.	Kriegstein A, Alvarez-Buylla A. The glial nature of embryonic and adult neural stem cells. Annu Rev Neurosci. 2009;32:149-84. doi: 10.1146/annurev.neuro.051508.135600. 19555289.

35.	Shimada IS, LeComte MD, Granger JC, Quinlan NJ, Spees JL. Self-renewal and differentiation of reactive astrocyte-derived neural stem/progenitor cells isolated from the cortical peri-infarct area after stroke. J Neurosci. 2012;32(23):7926-40. doi: 10.1523/JNEUROSCI.4303-11.2012. PMC3398807.

36.	Obernier K, Alvarez-Buylla A. Neural stem cells: origin, heterogeneity and regulation in the adult mammalian brain. Development. 2019;146(4):dev156059. doi: 10.1242/dev.156059. PMC6398449.

37.	Postuma RB, He W, Nunan J, Beyreuther K, Masters CL, Barrow CJ, et al. Substrate‐bound β‐amyloid peptides inhibit cell adhesion and neurite outgrowth in primary neuronal cultures. J Neurochem. 2000;74(3):1122-30. doi:

38.	Frost GR, Li Y-M. The role of astrocytes in amyloid production and Alzheimer's disease. Open biology. 2017;7(12):170228. doi: 10.1098/rsob.170228. PMC5746550.

39.	Söllvander S, Nikitidou E, Brolin R, Söderberg L, Sehlin D, Lannfelt L, et al. Accumulation of amyloid-β by astrocytes result in enlarged endosomes and microvesicle-induced apoptosis of neurons. Mol Neurodegener. 2016;11(1):38. doi: 10.1186/s13024-016-0098-z. PMC4865996.

40.	González-Reyes RE, Nava-Mesa MO, Vargas-Sánchez K, Ariza-Salamanca D, Mora-Muñoz L. Involvement of astrocytes in Alzheimer’s disease from a neuroinflammatory and oxidative stress perspective. Front Mol Neurosci. 2017;10:427. doi: 10.3389/fnmol.2017.00427. PMC5742194.

41.	Hegewald C, Alt R, Hetz S, Cross M, Acikgoez A, Till H, et al. Reduced oxygen stress promotes propagation of murine postnatal enteric neural progenitors in vitro. Neurogastroenterol Motil. 2011;23(10):e412-e24. doi: 10.1111/j.1365-2982.2011.01761.x.

42.	Shan Q, Zhuang J, Zheng G, Zhang Z, Zhang Y, Lu J, et al. Troxerutin reduces kidney damage against BDE-47-induced apoptosis via inhibiting NOX2 activity and increasing Nrf2 activity. Oxid Med Cell Longev. 2017;2017. doi: doi.org/10.1155/2017/6034692.

43.	Ma W, Wang S, Liu X, Tang F, Zhao P, Cheng K, et al. Protective effect of troxerutin and cerebroprotein hydrolysate injection on cerebral ischemia through inhibition of oxidative stress and promotion of angiogenesis in rats. Mol Med Report. 2019;19(4):3148-58. doi: 10.3892/mmr.2019.9960. PMC6423560.
